# Telling our stories: heroin-assisted treatment and SNAP activism in the Downtown Eastside of Vancouver

**DOI:** 10.1186/s12954-017-0152-3

**Published:** 2017-05-18

**Authors:** Susan Boyd, Dave Murray, SNAP, Donald MacPherson

**Affiliations:** 1 Faculty of Human and Social Development, University of Victoria, Victoria, BC V8W 2Y2 Canada; 2 c/o VANDU, 380 East Hastings Street, Vancouver, BC V6A 1P4 Canada; 3 Canadian Drug Policy Coalition, Centre for Applied Research in Mental Health and Addictions, Simon Fraser University, #2400 - 515 West Hastings Street, Vancouver, BC V6B 5K3 Canada

**Keywords:** Heroin-assisted treatment, Drug user groups, Community-based research, Overdose, Ethics, Activism

## Abstract

**Background:**

This article highlights the experiences of a peer-run group, SALOME/NAOMI Association of Patients (SNAP), that meets weekly in the Downtown Eastside of Vancouver, British Columbia, Canada. SNAP is a unique independent peer- run drug user group that formed in 2011 following Canada’s first heroin-assisted treatment trial (HAT), North America Opiate Medication Initiative (NAOMI). SNAP’s members are now made up of former research participants who participated in two heroin-assisted trials in Vancouver. This article highlights SNAP members’ experiences as research subjects in Canada’s second clinical trial conducted in Vancouver, Study to Assess Longer-term Opioid Medication Effectiveness (SALOME), that began recruitment of research participants in 2011.

**Methods:**

This paper draws on one brainstorming session, three focus groups, and field notes, with the SALOME/NAOMI Association of Patients (SNAP) in late 2013 about their experiences as research subjects in Canada’s second clinical trial, SALOME in the DTES of Vancouver, and fieldwork from a 6-year period (March 2011 to February 2017) with SNAP members. SNAP’s research draws on research principles developed by drug user groups and critical methodological frameworks on community-based research for social justice.

**Results:**

The results illuminate how participating in the SALOME clinical trial impacted the lives of SNAP members. In addition, the findings reveal how SNAP member’s advocacy for HAT impacts the group in positive ways. Seven major themes emerged from the analysis of the brainstorming and focus groups: life prior to SALOME, the clinic setting and routine, stability, 6-month transition, support, exiting the trial and ethics, and collective action, including their participation in a constitutional challenge in the Supreme Court of BC to continue receiving HAT once the SALOME trial ended.

**Conclusions:**

HAT benefits SNAP members. They argue that permanent HAT programs should be established in Canada because they are an effective harm reduction initiative, one that also reduces opioid overdose deaths.

As we complete this paper in April 2017, the Downtown Eastside (DTES) of Vancouver, in the province of British Columbia (BC), Canada, is experiencing the worst opioid overdose crisis in its history. Due to the unprecedented number of overdose deaths in the province (since 2012, illegal fentanyl-detected deaths have accounted for a steep rise in overdose deaths), in April 2016, a public health emergency was announced by Dr. Perry Kendall, the BC Provincial Health Officer [1, 2]. In 2016, there were a total of 931 overdose deaths in the province of BC, an increase of almost 80% from 2015 [2]. Thus far, the federal government has refused to declare a federal public health emergency, even though opioid drug overdose deaths have been rising in other areas of Canada too (as they are in regions of the USA). Following the release of the total overdose deaths in BC for 2016, the federal Health Minister announced that the government is assembling a roundtable of experts to consider expanded treatment options such as heroin-assisted treatment (HAT), hydromorphone, and slow release morphine [3]. Meanwhile, provinces, municipalities, and health authorities in Canada continued to work without increased funding from the federal government to stem the crisis.

Due to the lack of a full response by the federal government to the overdose death crisis, in September 2016, in defiance of federal law, activists set up two unauthorized supervised injection tents in the DTES of Vancouver. These community actions were followed up by the BC Minister of Health, announcing in December 2016, that rather than waiting for formal federal approval for safer injection sites, “overdose prevention sites” (small sites established in community services) would open in Vancouver and other areas of BC. Ten months into the public health emergency, the federal government announced in February 2017 that it has earmarking $10 million in health care funds to address the opioid crisis in BC. However, it is unclear how funds will be spent. Meanwhile, calls for expanded heroin-assisted treatment and hydromorphone have grown.

This article explores, through the voices of SALOME/NAOMI Association of Patients (SNAP), the benefits of HAT, the necessity for the immediate establishment of HAT and other alternative harm reduction programs throughout Canada, and the need to legalize and regulate currently criminalized drugs to stem the crisis.

This paper begins with a short historical summary of drug treatment and two clinical trials in Canada to contextualize our research, drawing on one brainstorming session and three focus groups with SNAP members conducted in late 2013 about their experiences as research subjects in a second HAT clinical trial in the DTES, the Study to Assess Longer-term Opioid Medication Effectiveness (SALOME). Also included are findings from ethnographic fieldwork over a 6-year period (March 2011 to March 2017) with SNAP members. SNAP is a unique peer-run independent drug user group made up of former research participants who participated in one or both HAT trials in the DTES.

This paper highlights SNAP’s ongoing advocacy for HAT, including their involvement in a constitutional challenge in the Supreme Court of British Columbia, to continue receiving HAT once their participation in the SALOME trial ended. In 2013, HRJ published SNAP’s (formerly NPA) first article about their experiences in the first HAT trial in the DTES [4]. Because the second HAT trial differed quite substantially from the first, and the social and political environment had also changed, SNAP set out to conduct a follow-up study of its members’ experiences.

## Background

As noted above, in order to contextualize the experiences of SNAP members, we provide a brief history of publicly funded drug treatment and two clinical trials in Canada. It may surprise readers to learn that Canada did not set up publicly funded drug treatment programs after heroin and other drugs were criminalized in the early 1900s. It was not until the late 1950s and 1960s that the first publicly funded drug treatment programs were set up in secure units in prisons, rather than in the community. These programs were abstinence based.

In 1959, the Narcotic Addiction Foundation of British Columbia (NAFBC) began to prescribe methadone to ease withdrawal for some of their patients [5]. This was possible because some of the legal restrictions enacted in the 1920s that made it illegal for doctors to issue a prescription for “non-medical” or addiction maintenance purposes to “known addicts” were finally lifted in the Narcotic Control Act of 1957 and 1961. Thus, physicians could begin to provide some alternative treatments. Following the dramatic increase in drug use among Canadians in the 1960s, the Canadian Commission of Inquiry into the Non-Medical Use of Drugs (the Le Dain Commission) was established in 1969. After completing its research and consultations, the Le Dain Commission recommended expanding public funded drug treatments and services and establishing methadone maintenance programs throughout Canada. The Commission also recommended that prison time for possession of criminalized drugs such as heroin should end [6].

Following the Le Dain Commission, drug treatment options expanded in Canada alongside increased criminal justice control [7]. However, abstinence-based programs predominated. Yet a change was brewing, and by the late 1980s and early 1990s, harm reduction was emerging in and outside of Canada as an alternative to abstinence-based models of treatment. In the DTES, one of Canada’s poorest urban neighborhood, harm reduction initiatives were seen by many as a practical tool to save lives [8]. The DTES has long been a place where residents actively come together to demand and make change, and they did so in the 1990s to implement drug policy reform [9, 10]. In the early and mid-1990s, the DTES experienced rising overdose deaths. In 1993, the Minister of Health and the Attorney General of BC responded by appointing the Chief Coroner, Vince Cain, to lead a task force inquiring into the rise of overdose deaths in the province. Following 8 months of consultations, the task force released its 1994 report, “The Report of the Task Force into Illicit Narcotic Overdose Deaths in British Columbia.” The report made clear that the “War on Drugs” was “an expensive failure” and linked prohibitionist policies to overdose deaths in the province ([11], p. vi). The Chief Coroner recommended expanded treatment and harm reduction programs and access to naloxone. The Chief Coroner also recommended that heroin-assisted treatment and the legalization of drugs be considered. In the meantime, the report recommended that the decriminalization of simple possession of all illegal drugs be immediately implemented [11]. However, most of the report’s recommendations were not put into action, and overdose deaths continued to rise alongside escalating HIV/AIDS and hepatitis rates.

The failure to respond to the crisis galvanized social activists into action. Demands included an end to drug prohibition, the creation of a federally sanctioned supervised injection facility in the DTES, and expanded harm reduction initiatives, including HAT [9, 10]. A few years after the Chief Coroner’s report, in 1997, the Vancouver/Richmond Health Board declared a public health emergency; yet a year later, overdose deaths and infection rates continued to rise [9]. Speaking at a press conference in August 1998, Bud Osborn, a long-time poet, activist, and co-founder of Canada’s first drug user union, the Vancouver Area Network of Drug Users, and then New Democratic Party Member of Parliament, Libby Davies, commented on the overdose crisis and offered concrete solutions: safer injection sites and heroin-assisted treatment so that people who used illegal opioids would no longer be vulnerable. Libby Davies argued:

“These deaths are preventable. It’s the responsibility of all levels of government to deal with the crisis. We ignore it at our peril” [12].

The harms stemming from punitive drug prohibitionist policies were highlighted by Bud Osborn, Libby Davies, and many others. Legalization of criminalized drugs was long advocated as a necessary policy directive to save lives [9]. Due to the efforts of activists, the City of Vancouver began to take steps. Spurred on by Mayor Philip Owen, alternative drug policies were seriously considered to stem the crisis. The City hosted several public events in 2001 and developed the Four Pillars’ approach to reduce the harms of drug use in Vancouver: prevention, treatment, harm reduction, and law enforcement. Expanded harm reduction programs, including the establishment of HAT and safer injection sites, were recommended in the subsequent report [13].

Unfortunately, the federal government of Canada ignored some of the key harm reduction strategies put forth by the Four Pillar’s approach prior to the overdose death crisis in Vancouver in the 1990s, including safer injection sites and heroin-assisted treatment. Needing federal approval, it took until 2003 for Canada’s first safer injection facility, Insite, to open in the DTES, and it was not until 2015 that a second smaller site was formally authorized at the Dr. Peter Centre in Vancouver. At the supervised injection and overdose prevention sites, not one overdose death has occurred [2]. In 2001, the federal government rejected a request by the Portland Hotel Society (PHS), a non-profit social, health, and housing agency that advances harm reduction approaches in the DTES, for legal permission to prescribe pharmaceutical heroin in Vancouver [3].

## Clinical HAT trials in Canada

Following Switzerland’s success with HAT in the 1990s, other European countries adopted similar models, providing a “rich data set on the feasibility, efficacy, safety and effectiveness of HAT” ([14], p. S151). However, rather than draw on early positive results and apply them in a Canadian context, in 2005, the first Canadian HAT clinical trial, North American Opiate Medication Initiative (NAOMI), opened in the DTES and Montreal [15]. Research participants in the NAOMI study were randomized into groups: one received injections of heroin (diacetylmorphine) or Dilaudid (hydromorphone) and the other received oral methadone. Each day research participants had to travel up to three times a day to the clinic to receive their doses. Prior to and following their dose, SALOME staff observes each participant for adverse effects.

Similar to international studies, NAOMI found that HAT proved to be a safe and highly effective treatment for people with chronic heroin addiction who have not benefited from other treatments, “including decreased use of illicit ‘street’ heroin, decreased criminal activity, decreased money spent on drugs, and improved physical and psychological health” [15]. However, the follow-up to the NAOMI study turned out to be unlike that of every other nation that has conducted a HAT trial. The Declaration of Helsinki guidelines developed by the World Medical Association to guide ethical medical research state:

[the] well-being of the individual research subject must take precedence over all other interests .… At the conclusion of the study, patients entered into the study are entitled to be informed about the outcome of the study and to share any benefits that result from it, for example, access to interventions identified as beneficial in the study or to other appropriate care or benefits .… Some research populations are particularly vulnerable and need special protection. These include those who cannot give or refuse consent for themselves and those who may be vulnerable to coercion or undue influence [16].

Nevertheless, despite these guidelines and the recommendations of the World Health Organization and UNAIDS 2011 report: *Ethical Engagement of People Who Inject Drugs in HIV Prevention Trials* [17] that clinical trial participants be offered continued treatment at the end of a trial if the treatment is found to be effective, NAOMI did not have such an exit strategy in place following the end of the trial. Research participants were therefore forced to discontinue treatment [4, 18].

Because a small number of research participants (24 participants) in the NAOMI trial received injectable hydromorphone and were not able to distinguish it from diacetylmorphine, NAOMI researchers decided to conduct a new trial. They pointed to the stigma associated with heroin as an obstacle; they argued that less stigma is attached to the synthetic opioid medication hydromorphone. Ignoring how HAT programs have been successfully set up in numerous diverse countries as well as the concerns surrounding hydromophone (a licensed pain medication, yet not licensed for opioid addiction treatment), in December 2011, another clinical trial opened its doors in the DTES, the Study to Assess Longer-term Opioid Medication Effectiveness (SALOME). The clinical trial recruited, once again, the most vulnerable of participants, long-term illegal opioid users, to compare hydromorphone to diacetylmorphine. The randomized clinical trial also compared the effectiveness of injection to oral doses of the medications after 6 months of treatment. All research participants injected their dose for the first 6 months; however, for the next 6 months of the study period, half of them were randomly switched to oral doses of the same drug [19].

Once again a permanent HAT program was not a component of the SALOME clinical trial, and participants exiting the study would be forced to discontinue treatment [19]. It should be noted that SNAP members are not opposed to hydromorphone in and of itself if it proves to be effective for *long-term* opioid maintenance. However, SNAP argues that hydromorphone should not replace HAT for those who benefit from the latter treatment. SNAP also questions the *assumptions* of the SALOME researchers that HAT was not achievable in Canada and their lack of effort to achieve this goal following the NAOMI study. SNAP also questions the establishment of another new trial (SALOME) that lacked an ethical exit plan. SNAP argues that rather than conducting another trial, diacetylmorphine should have been provided in a program, thus preventing harm.

## SNAP emerges

Responding to the failure of a permanent HAT program being established following the end of the first Canadian HAT trial (NAOMI), in January 2011, Dave Murray, who had been a research participant in the NAOMI trial, organized an independent, peer-run mutual support group to meet weekly at the Vancouver Area Network of Drug Users (VANDU) site in the DTES. VANDU is Canada’s oldest drug user union with a long history of activism. The peer-run group organized by Dave Murray was supported by VANDU. Initially named the NAOMI Patients Association (NPA), the group understood their unique status: at that time, they were the only Canadians or North Americans for that matter, to be recipients of HAT. All of the members of the newly formed peer group, NPA, had been research subjects in the NAOMI trial in the DTES at Crosstown Clinic [4]. To be clear, outside of having been a research subject in the NAOMI trial, the NPA is not affiliated with or supported by NAOMI or any other clinical trial. NPA is an independent peer-run drug user group. Similar to other peer-run drug user groups and unions around the world, NPA seeks to improve the lives of people who use illegal drugs and to advocate for change.

Almost a year after NPA was established, in December 2011, as noted above, another clinical trial, SALOME, was launched in the DTES. It too had no exit strategy or plans for establishing a permanent HAT program if the study found HAT to be effective [4]. NPA sought legal advice and also reached out to SALOME researchers/staff before they began treating research participants at Crosstown Clinic and invited them to a weekly meeting. At the meeting held in July 2011, NPA shared their study results and recommendations (from research that NPA had conducted with their own members about their experiences as participants in the NAOMI trial) with the SALOME team. NPA also communicated ways to improve the experiences of research participants entering the new clinical trial. Yet the one main issue, the lack of an ethical exit strategy for research participants at the end of the SALOME clinical trial, was not fully heeded. Once the SALOME trial was underway in the early summer of 2013, with many NPA members as research subjects, the NPA group voted to change their name to SALOME/NAOMI Association of Patients (SNAP) to better reflect their membership. SNAP’s mission statement below sets out their goals.

### SALOME/NAOMI ASSOCIATION of Patients (SNAP)

SNAP is a unique group of people who were participants in the NAOMI and/or SALOME heroin-assisted therapy (HAT) clinical trials in Vancouver, BC. We are an independent group dedicated to supporting each other and educating peers, researchers, government, and the public. We advocate for the human rights of people who use opiates, the establishment of permanent and less medicalized HAT programs in Canada, and an end to drug prohibition.

SNAP meetings include from 10 to 40 members each week. Women make up about a quarter of SNAP membership. The majority of SNAP members are receiving social welfare or disability benefits. Members are drawn from the greater Vancouver area; however, the majority of members reside in the DTES. Meetings are held each week on Saturdays and begin with a round of introductions by members, followed by an agenda made by members that list issues to report on or to discuss. At the end of each SNAP meeting, a moment of silence is held in memory of all friends and family who have died. Increasingly, the overdose crisis has become a focus of SNAP members as the death rate climbs. Since 2011, many cities in BC have been hit hard by the overdose death crisis; however, Vancouver has experienced the most deaths and SNAP members have lost friends and family [2].

## Methods

In March 2011, the lead author was invited by SNAP to attend weekly meetings and to collaborate with the group on a research project. The SNAP group determines the research agenda, and the lead author helps to facilitate the collaborative research process and the dissemination of results at public forums and conferences. Field notes are recorded at each weekly meeting. In 2011, neither the lead author nor the SNAP group envisioned collaborating for over 6 years. However, further advocacy by SNAP, and outside events, led to the group’s decision to conduct further research to capture the experiences of their members. Similar to the group’s earlier collaborative research, conducted in 2012, SNAP’s current research draws on research principles developed by drug user groups, such as VANDU, and critical methodological frameworks on community-based research for social justice [4, 20–23]. As was the case with their early research, the SNAP members drew from a poem written by Sandy Cameron, *Telling Stories*, to guide their research [24].

**Telling Stories**

We need to tell our own stories.

If we don’t tell our stories,

People with power

will tell our stories for us.

And we won’t like what they say.

When we tell our stories,

we reach out to each other

And build community.

Sandy Cameron was a long-time activist and poet in the Downtown Eastside. His poem exemplifies the spirit of the SNAP research project, the goal to tell “our own stories” about HAT trials in the Downtown Eastside of Vancouver, the quest to set up HAT programs and ultimately end drug prohibition.

In order to better understand SNAP experiences as they participated in the second clinical trial in the DTES, with the consent of the group, the lead and second author conducted a brainstorming session with members to identify specific areas to investigate, followed by three focus groups conducted in 2013 with a total of 17 SNAP members (12 men and 5 women). In addition, agenda and ethnographic field notes from weekly meetings and events from March 2011 to March 2017 were examined by the lead author to provide further context about events related to HAT and the SNAP group. The research project received ethics approval from the University of Victoria, BC. All SNAP members were granted confidentiality and anonymity and signed a consent form prior to participating in the research. The focus groups were recorded and transcribed by the lead author, and all identifying information was removed; the transcriptions were then brought back to SNAP, and further discussion led to a coding schedule.

The transcripts were analyzed drawing on a method of comparison and questioning; thus, themes were identified not only through the brainstorming session and focus group questions but also from the data. In telling their story, seven major themes emerged from the brainstorming and focus groups: life prior to SALOME, the clinic setting and routine, stability, 6-month transition, support, exiting the trial and ethics, and collective action.

To facilitate the collaborative research process, a hard copy of identified themes, analysis, and findings were distributed to SNAP members at weekly meetings and read out loud and discussed over a 4-month period. SNAP members provided feedback, and the lead author incorporated their comments each week until an approved draft paper was complete. The back and forth process with SNAP members was laborious; however, during each stage of the process, collaboration was assured. The collaborative writing process began again in 2016 when SNAP made the decision to revise the paper in order to include a discussion of more recent events. The themes identified above are expanded upon in these pages.

The research approach undertaken made visible the diverse experiences of the members of SNAP and is attentive not only to the research process but also to input by participants and communication of the results to the wider public. In so doing, SNAP’s research is relevant to policy making. The findings below capture some of the shifts, tensions, activism, and hopes of SNAP members for ethical harm reduction services and drug policies.

## Results

### Life prior to SALOME

A number of the SNAP focus group participants spoke about their life prior to entering the SALOME trial and the difficulties they faced, including obtaining money to support their use of a criminalized drug. One male participant stated:

My habit was going wild .… I was spending a ton of money on heroin and on opiates — of all kinds, actually.

The SALOME trial recruited a total of 202 participants between December 2011 and December 2013. However, the start and exit date for participants differed. For many, the start date was months away from their recruitment. One male participant explained that his health was deteriorating prior to getting into SALOME:

My life .… was becoming unglued before I could get on the SALOME project. I found out it was going to take me about 11 months before I could get on the program.

Another participant pointed out that he sold drugs and stole to support his addiction to heroin:

I found out about the program at the Pigeon Park Savings Bank .… and they told me they would get ahold of me. It was a year and four months before I got contacted. In that time, you know, I sold drugs, I dealt, I stole. I did whatever I did to get the money for dope.

One woman who had also been a NAOMI patient and was now a SALOME patient reported on the period after the first clinical trial ended and prior to the start of the SALOME trial:

[It was] tough, it cost me a lot of money. Ate all my [welfare] check. I am left with maybe $100. each month for food, or whatever.… All you focus on is how am I going to get the money, you don’t have time for anything else.

For most of the SNAP participants, it was difficult to avoid participating in illegal activities prior to participating in SALOME clinical trials, even if this meant obtaining heroin on the illegal market. Another woman explained why she wanted to be a SALOME patient:

I didn’t want to be walking the streets. I didn’t want to be selling stuff. I didn’t want to be stealing stuff. I didn’t want to be stealing from my dates and getting hurt because of it, because when I’m sick .… you’re hurting.

Her comment is especially potent given gender inequality and ongoing systemic violence against poor women in and outside of the DTES, and more specifically, gender violence against Indigenous women in Canada as a “durable feature of colonial power relations” ([25], p. 7; [26]). For the majority of the participants in the three focus groups, a 13-month reprieve while they were registered as a patient in the SALOME clinical trial was all that mattered. One other participant explained the situation:

You’re sick. It’s free dope. Plain and simple, it’s free dope. That’s what they were after. You know, they don’t have to worry about their habit for a year or whatever.

Some SNAP participants paid little attention to the details, including release of information forms (to physicians, Vancouver Police Department, PharmaNet, Ministry of Employment and Income Assistance, etc.) and consent forms that they signed for the SALOME study. One participant said:

I was so sick I didn’t read all of the rules and regulations .… You know, it’s 30 pages long and I just signed everything because I was so sick.

Regardless of the rules and the parameters of the trial, the participants hoped to benefit from their participation in the study.

## The clinic and routine

Once participants discussed getting into the SALOME trial, they turned their attention to getting to and from Crosstown Clinic in the DTES, where, like the NAOMI clinical trial, the SALOME trial was conducted. Each day participants had to travel two or three times a day to the clinic to receive their doses of heroin (diacetylmorphine) or Dilaudid (hydromorphone). Following their dose, research participants had to leave the clinic; there was no place (such as a kitchen or living room space) set up for them to spend more time at the clinic after they were observed.

When asked what the clinic looks like, a woman participant recommended that Crosstown Clinic apply a “bit of paint” and “make it more bright.” A few weeks after the focus groups ended, during their weekly Saturday meeting, SNAP members watched a Danish documentary titled *Anyone for Coffee and Heroin?* The film documents the first year of a heroin-assisted treatment program (not a trial) called Poppy in Denmark. The documentary follows the lives of both staff and patients at Poppy. SNAP members noted that the Poppy clinic stood out in stark contrast to Crosstown Clinic where the SALOME clinical trial was conducted. Whereas Crosstown Clinic is small and sterile, the Poppy clinic is informal and welcoming, bright and airy, with sun streaming in from large windows. At the Poppy clinic, the walls are painted white and covered in large colorful abstract paintings. SNAP members were astonished to see that patients at the Poppy clinic had all day access to a kitchen, meals, a living room area, a gym, and a sun deck on the roof of the clinic. The patients at Poppy also had access to social and legal support, housing, and therapy. In the documentary, housing in Denmark is depicted as consisting of one-bedroom apartments rather than single-room occupancy (SROs) or small studio apartments so favored for the poor by all levels of government in Canada [27].

The participants spoke about the security and safety measures, the routine at Crosstown Clinic, and the administration of their doses during the trial. One woman explained that every time they arrived for their two or three doses a day, they were buzzed in through two locked secure doors:

We have 10 minutes of an open-door system where if you’re there you get 10 minutes from – like, if your dose is at 12:00, you get 12:00 to 12:10 to get in, and if you’re even a minute late you’re not allowed in. You can flex another time by phoning a number or showing up at the door, but sometimes you can phone at 10 o’clock in the morning, and you won’t get a morning session .… So if you miss your time, you could possibly miss that whole session and you’ll only get two a day, two – so that’s two shots.

The focus group participants also explained that once they were buzzed through the door, they had a 5-min waiting period where they were then evaluated by SALOME staff in a small room with some tables and chairs before they were allowed to go for their injection. Then, they moved to pick up their dose, and they had 7 min to inject it (in the first 6-month phase of the trial, participants injected their dose). Once the participants received their dose, they moved into another room where they had to stay for at least 20 minutes before leaving the building. Thus, the trial clinic setting was highly regulated.

Because the SALOME trial was a double-blind study, neither the participants nor the researchers knew which drug was being administered (heroin or Dilaudid). However, participants in the trial knew that there was a 50% chance of receiving heroin [19, 28]. At the focus groups, participants brought up that they were uneasy not knowing which drug they were taking every day. One woman noted:

It’s kind of creepy not knowing. You know, and it makes you wonder .… Our imaginations can be quite explicit.

Most participants stated that they had a right to know what they were putting into their bodies, if not during the trial, then as soon as they exited the trial.

## Stability and 6-month transition difficulties

SNAP members discussed the stability of being a SALOME participant, how their overall well-being improved. One recent SALOME patient noted:

My life is starting to become more manageable and everything .… and I’m only two and a half months into it .… I’m putting on weight, that’s one thing. I’m eating better.… It’s stabilized my life .… I don’t wake up in the morning having to figure out what crime I’m going to do to pay for my drugs .… and I’m actually looking for other things in my life, like even going swimming, leisure and stuff like that. … And this is only at the start.

Another man who was in the early months of the trial commented:

I don’t get sick. I sleep all night. I don’t do crimes. That’s really good.

One woman who was entering the last stages of the study explained to the focus group:

I have had a year reprieve, sanity for a year.

Many focus group participants were worried that the stability they achieved during the first 6 months of the trial would be compromised if they were randomly switched to oral doses rather than injection when they reached the 6-month transition period of the study. SALOME participants injected their dose for the first 6 months; however, for the next 6 months of the study period, half of them were randomly switched to oral doses of the same drug and for many, the switch was very difficult.

One woman stated:

Well, I got switched, and I never thought it would be, you know, as hard as it is, but I’ve actually gone to — you know, I don’t like to say this, but I’ve actually gone to some of my old ways just because, like, it’s not what I want, you know.

For some participants in the focus group, being switched to oral from injection was problematic and led to them using illegal heroin again and injecting the drug:

While I was on injection, I didn’t use — after the first month I didn’t use any [street] heroin. I didn’t use any powder cocaine. After about two months on the oral Dilaudid, the hydropmorphone, I found myself starting to inject street drugs again.

One woman who was switched from injectable to oral stressed that:

Six months of injectable is not enough, it should have been a year. You can’t get proper results in six months. In NAOMI it was for a year.

The participant refers to the fact that participants in the first HAT trial in Canada, NAOMI, were provided injectable heroin for 1 year prior to exiting the study. The NAOMI participants were not required to switch from injection to oral. Another focus group participant and SNAP member discussed how he was not able to remain stable when he was switched over to oral doses at the 6-month period. He quit the trial:

I’m not on the program. When I got switched over to oral, I just couldn’t make it work. So for all the benefits and things like that that I got from it while I was on the injection side, I’ve got to say after coming off it it’s like walking away from a huge Methadone habit more or less cold turkey and trying to find enough of whatever opiates you can to get better or try to get better.

Another participant stated that:

I was on injection for six months, and then I was told I had to go onto oral. I don’t like the oral. It doesn’t satisfy me. I still am sick every day.

Another participant concluded that the 6-month transition should not have been enforced and that research participants should have choices:

If I had to write the study over or help them write the study I would have given a bit more choice involved at those six months. I don’t think it would have screwed up their — what they wanted to learn out of it, whether Dilaudid actually was a good substitute for injectable Dilaudid compared to injectable heroin, oral heroin or oral Dilaudid.

He ended with this powerful statement:

We want people that use opiates to have a choice .… and part of it [SALOME] runs counter to that objective, that I still don’t have choice in this. I’m told what I’m getting and I’m told what I’m doing.

In Canada, research participants who participate in HAT clinical trials have had little choice about the treatment they are offered and the needs expressed by research participants are often ignored. For some research participants in the SALOME trial, the switch from injection to oral doses and the lack of choice about routes of administration were problematic.

## Ruin the science by helping people out

Participants talked about receiving social support while participating in the SALOME trial at Crosstown Clinic two or three times a day over a year. They noted that most of the staff and their doctors at the clinic were very supportive and caring. However, the biggest critique by the participants was that social supports to improve people’s lives were not offered by SALOME, especially at the beginning of the trial, because it would, as one participant noted:

Ruin the science by helping people out.

The SALOME trial measured the efficacy of heroin and Dilaudid, and more meaningful supports (educational, legal, employment, and economic) were not included in the study proposal. This was despite the fact that SNAP members pointed to the necessity of such supports when they met with SALOME researchers prior to participating in the new trial. Participants discussed their lack of housing; one participant explained that he lived in an SRO for over 10 years:

I haven’t been able to get social housing of any form.

Another participant said that she “lives in a crappy hotel now .… horrible place, bug infested.” SALOME had not been able to help her find housing even after 12 months. However, over time, many SNAP members were able, oftentimes with the help of SALOME staff, to find more suitable social housing.

Canadian scholar Dara Culhane made clear in her own research that although health and social science research in the DTES has expanded since the 1990s, the social conditions of people living there remain the same or have even worsened [21]. It is a tension and an ethical question that shapes every research project conducted with vulnerable and marginalized people/communities in and outside of the DTES.

Other participants explained that they needed more support during and at the end of their participation in the SALOME trial or prior to their transition period. One man expressed that he felt like a “prisoner,” because he did not know what would happen to him at the end of 12 months when he was cut off his treatment and began his transition back to conventional treatment. And a woman asked:

What’s going to happen to us after, you know?

Another man asked who will “help us out when we finish, you know?”

## Exiting the trial and ethics

As noted above, unlike the NAOMI trial, half of the participants in SALOME were randomly selected for a change in the administration of their dose, from injection diacetylmorphine or hydromorphone to an equivalent oral dose 6 months after entering the trial. Following 12 months of treatment, SALOME patients had 3 months prior to their exit from the study to transition back to conventional treatments, including treatment options that had not worked for them in the past. As SNAP members neared the end of their year in the SALOME trial, they were anxious about their future. Some members had been NAOMI patients and thus had already experienced a tumultuous period following the end of that clinical trial.

Came off of NAOMI and didn’t do too well .… [In SALOME] I’m at about 11 months of injection. I’ve been called in by a doctor and recently told, get prepared for the end because the end is near.

All SALOME patients faced this dilemma because the clinical trial did not include an ethical exit strategy for its patients outside of transitioning to conventional treatments that had failed them in the past. However, SNAP and other advocates, such as VANDU, Portland Hotel Society, and other groups and individuals in BC, advocated for change in SALOME policy. In April 2012, the NPA consulted with Pivot Legal Society in Vancouver to seek support in their quest for HAT. “Pivot’s mandate is to use the law to address the root causes of poverty and social exclusion” [29]. Pivot and SNAP collaborated in efforts to inform Providence Health Care Society and other stakeholders of the ethical and legal issues with both the NAOMI and SALOME studies. By early 2013, Providence Health Care Society (PHCS) (BC’s public health care provider) and some Crosstown Clinic doctors also began to seek a more feasible exit strategy for research participants when they completed the SALOME trial.

In May 2013, Providence Health Care Society announced that patients exiting SALOME would be temporarily offered Dilaudid at Crosstown Clinic. SNAP members were relieved that they did not have to go back to failed treatment approaches when they exited the trial. But they were also wary of remaining on Dilaudid for any length of time because it is not a licensed drug and it has never been tested *long term* for maintenance purposes or the treatment of opioid addiction. In contrast, over the last 20 years, numerous international studies demonstrate that heroin-assisted treatment is a proven, effective treatment [14, 15, 30]. At the time that the focus groups were conducted with SNAP members in 2013, the SALOME trial results were not yet published. Unfortunately, the first, and to date the only, SALOME paper published drawing from the SALOME trial results only provided findings from the first 6 months of participation by patients. Thus, the 2016 SALOME paper did not include findings from the 6-month transition period from injection to oral doses nor the 6-month period following the transition period or the exit period [19]. After Providence Health Care Society announced that they were considering providing oral hydromorphone for SALOME patients exiting the trial, one participant stated:

The thing is what we have —when they’re talking about giving us oral Dilaudid at the end of the study, you know, and offering us that as if it’s a big prize — they’re offering us a drug that’s never been tested on human beings as a treatment for addiction .… I mean, Dilaudid has been used as a pain medication for many years, but it’s never been used as a maintenance drug where they’re going to continue for years and years and years and years of using it .… I don’t think it has ever been tested.

Because hydromorphone is not licensed for addiction treatment, SNAP members remained concerned about the negative impact the drug may have long term and argued that effective drugs, such as diacetylmorphine, which is proven to be effective long term for the treatment of opioid addiction, were being overlooked by addiction specialists in Canada. Focus group participants also noted that many participants, as noted above, did not thrive on oral doses; injecting their dose was a more stabilizing option for them. Thus, they worried that by providing oral doses of Dilaudid, more participants would suffer unnecessarily.

One woman explained that when she exited the study, she was offered oral Dilaudid, but it did not work for her:

I am doing [street] heroin every day now. I would like to get [legal] injectable heroin long-term. My life was so much better then.

Focus group participants also spoke about the short length of the SALOME trial and the need for a permanent HAT program. One participant noted:

When I read the whole thing, what it really says, Study to Assess Long-term [Opioid Medication Effectiveness] — what do they mean by long-term? There’s no long-term in this when they’re talking about one year.

Another male participant responded, “Exactly.”

The SNAP members who had been research participants in both trials pointed out that much of their frustration and their fears related to not knowing what drug they were prescribed, having no control over the length of their time receiving HAT, and coping with the rigid routine at the clinic. They felt that these issues could have been addressed to better accommodate ﻿participant’ needs. They also pointed out that early on the SALOME researchers could have taken their recommendations for change more seriously, especially following the group’s public forum in November 2011 and the release of their first research report in February 2012. The lack of a feasible exit strategy once again weighed heavily on the focus group participants and all recommended the setting up of a permanent HAT program. SNAP argues that ethical exit strategies and the establishment of permanent HAT programs should have been built into both the NAOMI and SALOME clinical trials.

## Collective action and the future

During the focus groups, SNAP participants discussed recommendations for future programs and drug policy in general. SNAP members discussed the benefits of a permanent and flexible HAT program.

I think there should be a program I mean, like, why have another stupid, you know, trial thing and that? Because, I mean, Denmark has a program because of NAOMI. Sinful, you know. Why don’t we?

If there was a permanent program .… at the person’s own speed. Like, if you didn’t want to get off the injectable side you could stay in the injectable side .… You know, you should be there [in HAT] until you .… think you can leave. Like, if you want to be on it for five years you should be on it for five years.

The focus group members also spoke about HAT programs around the world, where patients could stabilize and receive treatment without fear of being denied a beneficial medicine.

One participant brought up a larger factor that shapes their lives: drug prohibition or the war on drugs:

The way I look at it is it’s not the drug that ever caused me problems; it’s the [drug] war on being able to maintain it, you know, like, you have to have eyes in the back of your head, you know. I mean, it’s not the drug.

A core goal of SNAP members is to end drug prohibition. They understand that drug prohibition has led to a range of harms including punitive laws that criminalize heroin and other opioids, punitive drug treatment models, and legal and social discrimination against people who use criminalized drugs [31]. Drug prohibition makes it difficult for alternative treatments such as HAT to be put into place and shapes the lives of the members of SNAP. One member who dropped out of the SALOME trial after a few months because the drug he was prescribed did not work for him stated his life is a lie because of prohibition and the strict regulation of the drugs he needs to be well:

My whole life’s a lie to my doctor. I mean, like, I’ve made up all this bullshit to him so I can get my pills early this week, nah, nah, nah, you know what I mean? And it’s all crap.

Another SNAP member said:

I’d say the doctor should have everything in his tool bag and recognize the fact that everyone’s different .… what will work for me might not work for you.

Another SNAP member stated that the “powers that be” needed to “smarten up*.*” The member agreed that one way to inform the “powers that be” is as follows:

We’ve got to get our stories out there, our stories — and personalize the thing .… and attach faces, stories and real-life situations to the statistics.

Since December 2011, SNAP (formerly NPA) has fought for the human rights of their members despite the unequal power relations between SALOME researchers and patients. Yet, the *voices* of SNAP members are collectively powerful:

Can I just add right here .… the biggest thing we’ve tried to stress from the beginning, is to be independent from the powers that run SALOME. We want to be a patient’s voice that’s completely a patient’s voice. The ethics in any addictions study are difficult to deal with just because of the fact of the population, the difference in the power that’s there.

Another man commented that he attends meetings because of what SNAP has accomplished and that the group’s advocacy is important to him:

I think I come [to SNAP meetings] because of what the group has accomplished so far. I want to — I know it can accomplish more .… I just would really, really like to see a heroin treatment program in Vancouver.

His statement recognizes the many dimensions of SNAP’s advocacy. This work includes setting up and participating in public forums on HAT, conference presentations, supporting and participating in the launch of a Charter Challenge, reports, journal articles, letter writing campaigns, and meetings with health practitioners, government officials, researchers, the media, and the public. All these initiatives are undertaken by SNAP in an effort to establish permanent HAT programs in Canada (in contrast to clinical trials), to protect the human rights of people who use criminalized drugs and to end drug prohibition. This comment by the participant quoted above provoked the dialogue below about SNAP activism:

P1: These [SNAP] groups, it’s the first time in my life I’ve ever witnessed people, you know, working towards, you know .…

P2: a bunch of fucking junkies got something done.

P1: Yeah, I mean, it’s not the drug that fucks us up; it’s the fucking lifestyle that we wind up living to, you know .… I mean, you know, it’s so simple. It’s a no-brainer, but .… for me to go and tell one person that and hope they listen .… [it] is not going to change anything; the only way anything is going to change is by doing what we’re doing.

Marginalized people who use heroin are often framed as a social problem (as addicts, criminals, and pathological). However, members of SNAP understand their lives quite differently. SNAP members are negatively impacted by a range of structural factors, including drug laws and policies stemming from them, policing and criminal justice, gendered violence, colonialism, and neoliberalism. Yet with few resources outside of the support of VANDU, they are activists striving for change. Taking SNAP members’ comments to heart, the group continues to challenge the powers that be and to provide its members and the public with alternatives to clinical trials, unethical and punitive drug treatment, and current drug policy.

## Discussion

### Continued advocacy and legal challenge

By 2013, SNAP became hopeful about change stemming from their activism because Pivot Legal Society, Portland Hotel Society, and finally the local health authority, and addiction and ethics specialists supported HAT treatment. Providence Health Care Society and some of their doctors at Crosstown Clinic also understood their plight and with support submitted Special Access Requests for patients who would benefit from HAT after they exited the SALOME trial. The Special Access Programme (SAP) was set up by Health Canada to allow physicians treating patients with serious and life-threatening conditions and, for whom other conventional treatments have failed or are unavailable, to offer a medication that is not otherwise available in Canada.

On September 20, 2013, SNAP members heard that Health Canada approved 16 applications submitted by Crosstown Clinic physicians to the SAP for SALOME participants to receive injectable heroin for 3 months after exiting the trial. However, shortly after the approval, a widely circulated statement by Rona Ambrose, then federal Minister of Health, was sent to the media reprimanding the SAP’s decision to approve HAT and stating that the minister would close the “loopholes” in the federal regulations. Drawing from the statement by the Minister of Health, a message was also sent out by the Conservative Party of Canada, titled: “Stop giving heroin to addicts.” The message stated: “Drugs like heroin tear families apart, promote criminal behavior, and destroy lives .… drug treatment programs should be focused on ending drug use — not giving illicit drugs to drug addicts” [32]. These deeply discriminatory words by the then Health Minister and the Conservative Party of Canada’s majority government at that time are disturbing because international studies on HAT, the NAOMI findings, and SNAP/NPA research make clear that providing legally prescribed unadulterated heroin to people addicted to the substance is proven to be beneficial for this small population.

The SAP approved another five HAT requests on September 27, 2013, thus attempting to address the needs of 21 patients. However, on October 3, 2013, the federal government announced the changes to the federal regulations making diacetylmorphine (heroin) a restricted substance under the Food and Drug Act and, thus, no longer available through Health Canada’s SAP. On November 13, 2013, five plaintiffs, Dave Murray (the founder and meeting facilitator of SNAP), Douglas Lidstrom, Larry Love, Charles English, and Deborah Bartosch (four of the plaintiffs are long time members of SNAP, all former SALOME patients), along with co-plaintiff, Providence Health Care of BC, and their lawyers, filed a constitutional challenge in the BC Supreme Court to overturn the federal government’s decision to prevent further Special Access Requests for HAT. They argued that the new federal regulations infringe on the Canadian Charter of Rights and Freedoms for SALOME patients and should be struck down by the courts. Because the Charter case was not scheduled to be heard until the fall of 2016, BC Supreme Court Chief Justice Hinkson granted an injunction in May 2014, until the case could be presented [33]. In the meantime, former SALOME research participants could receive HAT at Crosstown clinic if their SAP application was successful. At Crosstown Clinic in the DTES, around 150 people are now receiving heroin (SAP applicants) or hydromorphone through the interim program (supervised injectable opioid-assisted treatment) [34].

Another significant event in 2015 also affected SAP applicants and their access to HAT. In the fall of 2015, following a federal election, the Liberal Party of Canada formed a majority government. The former Conservative government, led by Stephen Harper, vehemently opposed HAT and harm reduction initiatives such as supervised injection facilities and had waged a 10-year law and order campaign enacting more punitive drug laws and discriminatory policies. In September 2016, the Liberal government overturned the former government’s policy on SAP applications and reinstated the former policy. Thus, physicians on behalf of their patients can submit SAP requests for HAT again, and Crosstown Clinic is hoping to expand in 2017 to allow more patients to receive HAT. Because the old SAP policy was reinstated, the Supreme Court challenge will not be heard.

Today, many SNAP members are receiving HAT or hydromorphone at Crosstown Clinic in the program set up there. However, unlike other HAT patients in programs around the word, for those receiving HAT in Canada, an initial SAP application was submitted by their physician and approved by Health Canada. Every 6 months, a SAP renewal application must be submitted to Health Canada. In addition, it is a time-consuming process that is fraught with tension for the patients because of the many hurdles to have legal heroin shipped to Canada from Europe, delays by Health Canada, and the possibility that the request will be denied. SNAP is now conducting its third research project to capture the voices of those SNAP members who are now patients in the program at Crosstown Clinic. The stability of being a patient (not a research subject) in a HAT program, even one that was set up as an interim program, has been a positive and life-affirming experience for SNAP members. As well, SNAP members praise Crosstown Clinic staff for their support. They also praise Crosstown Clinic physicians such as Dr. Scott MacDonald and Dr. Cheryl McDermid as unsung heroes, for their compassion in submitting SAP applications for HAT for their patients and for their advocacy of HAT as an effective opioid treatment for those who can benefit. One SNAP member noted that he is thankful that he is a patient in the HAT program at Crosstown Clinic, because it has protected him during the overdose death crisis in BC:

That’s why it’s time to get more people in [the HAT program], you know, especially with everybody OD-ing on this crap that’s out there now .… Fentanyl, yeah. That’s scary, you know. I am really thankful I am not out there taking my chances like that.”

SNAP continues to advocate for permanent flexible HAT programs and the reclassification of diacetylmorphine, so that physicians in Canada can more easily prescribe it. Internationally, heroin-assisted treatment continues to be advocated for chronic opioid users in numerous countries, including the UK, Switzerland, Denmark, Germany, the Netherlands, and Spain [14, 30].

Of course, the difficulties of setting up permanent HAT programs, unnecessary drug overdose deaths, and the marginalization and criminalization of people who use opiates and other drugs without a prescription are products of drug prohibition. In addition, delays in establishing life-saving harm reduction programs like HAT and the labyrinth of punitive treatment modalities forced on people who use criminalized drugs can only be accomplished under drug prohibition. Sadly, for those most affected, drug prohibition and a reliance on criminal law, abstinence-based treatments, and limited harm reduction programs fail to reduce drug overdose deaths [9, 10, 31].

## Conclusions

This collaborative study highlights the experiences of SNAP members. All of the SNAP members were research participants in the SALOME clinical trial conducted in the DTES of Vancouver. Rather than be invisible, SNAP set out to tell their story, about being research subjects in a clinical trial and their advocacy for HAT. Due to the small number of participants in SNAP’s research, the findings may not be applicable to other drug user groups or people who are prescribed HAT outside of Canada. However, the findings highlight how participating in the SALOME clinical trial impacted the lives of SNAP members. The SNAP members assert that HAT benefits them and saves lives. In addition, the findings reveal how SNAP member’s advocacy for HAT impacts the peer-run group in positive ways.

Some of SNAP’s recommendations are reflected in a recent 2017 Coroner’s investigation into a fentanyl overdose death of a young man in a treatment centre in BC. The Coroner made 21 recommendations, including the following: “diacetylmorphine and hydromorphone treatment programs for chronic opioid users,” supervised consumption sites, and naloxone [35].

In contrast to the cumbersome SAP application process for HAT, described earlier in this article, in April 2017, Health Canada announced a new regulatory process to stem the opioid crisis and other public health emergencies or pandemics in Canada. Health Canada’s new process will allow the importation (from approved countries) and use of medications not yet authorized in Canada, such as heroin for HAT. Public health officials will send a request to Health Canada for bulk quantities of the drug. On approval, the medicine will be shipped and made available for the treatment of patients at clinics and other authorized sites.

Given that in 2016 almost 90% of all overdose deaths in BC occurred inside a residence and that no deaths have ever occurred at supervised injection sites or HAT trials in Canada, the establishment of HAT programs together with expanded supervised injection sites is crucial [2]. The Coroner’s other recommendations are also crucial, such as the recommendation for expanded access to naloxone. Given the recent findings of the Coroners Service of BC on overdose deaths and fentanyl detected with other substances [1, 2], there is also a need to scale up other harm reduction programs, including managed alcohol programs, the establishment of stimulant programs for individuals who use cocaine and methamphetamines, and the implementation of programs that allow for more flexibility in the administration of doses, such as injection, oral, smoking, and sniffing. Equally important, representatives from drug user groups should be at the table as new harm reduction programs are envisioned and implemented so that the services meet the needs of those most affected. In addition, peers from drug user groups can support research participants, helping them to negotiate study information and consent forms.

Finally, SNAP calls for the legal regulation of all criminalized drugs. Legal regulation is not the same as a free market approach; rather, it means that depending on the drug in question, diverse regulatory models with attention to human rights, social inclusion, and public health can be applied. This policy change would guarantee that the quality and quantity of all drugs are assured and that social and legal discrimination of people who currently use criminalized drugs ends. Given the overdose crises in and outside of British Columbia today, this might be the right time for a clean break from prohibitionist policy.

Thus, for SNAP, the fight continues.

## Abbreviations

BC: British Columbia
DTES: Downtown Eastside
HAT: Heroin-assisted treatment
HRJ: Harm Reduction Journal
Le Dain Commission: Canadian Commission of Inquiry into the Non-Medical Use of Drugs
NAFBC: Narcotic Addiction Foundation of British Columbia
NAOMI: North American Opiate Medication Initiative
NPA: NAOMI Patients Association
PHCS: Providence Health Care Society
SALOME: Study to Access Longer-term Opioid Medication Effectiveness
SAP: Special Access Program
SNAP: SALOME/NAOMI Association of Patients
SRO: Single-room occupancy
VANDU: Vancouver Area Network of Drug Users
