# Erratum to: Telling our stories: heroin-assisted treatment and SNAP activism in the Downtown Eastside of Vancouver

**DOI:** 10.1186/s12954-017-0161-2

**Published:** 2017-06-09

**Authors:** Susan Boyd, Dave Murray, SNAP, Donald MacPherson

**Affiliations:** 1 Faculty of Human and Social Development, University of Victoria, Victoria, BC V8W 2Y2 Canada; 2 c/o VANDU, 380 East Hastings Street, Vancouver, BC V6A 1P4 Canada; 3 Canadian Drug Policy Coalition, Centre for Applied Research in Mental Health and Addictions, Simon Fraser University, #2400 -515 West Hastings Street, Vancouver, BC V6B 5K3 Canada

## Erratum

“Upon publication of the original article [1], it was noticed that there was an error in the last sentence of the first paragraph. The sentence should read: Prior to and following their dose, NAOMI staff observes each participant for adverse effects.”
